# An Improved Proportionate Normalized Least-Mean-Square Algorithm for Broadband Multipath Channel Estimation

**DOI:** 10.1155/2014/572969

**Published:** 2014-03-20

**Authors:** Yingsong Li, Masanori Hamamura

**Affiliations:** Graduate School of Engineering, Kochi University of Technology, Kami-shi 782-8502, Japan

## Abstract

To make use of the sparsity property of broadband multipath wireless communication channels, we mathematically propose an l_*p*_-norm-constrained proportionate normalized least-mean-square (LP-PNLMS) sparse channel estimation algorithm. A general l_*p*_-norm is weighted by the gain matrix and is incorporated into the cost function of the proportionate normalized least-mean-square (PNLMS) algorithm. This integration is equivalent to adding a zero attractor to the iterations, by which the convergence speed and steady-state performance of the inactive taps are significantly improved. Our simulation results demonstrate that the proposed algorithm can effectively improve the estimation performance of the PNLMS-based algorithm for sparse channel estimation applications.

## 1. Introduction

Broadband signal transmission is becoming a commonly used high-data-rate technique for next-generation wireless communication systems, such as 3 GPP long-term evolution (LTE) and worldwide interoperability for microwave access (WiMAX) [1]. The transmission performance of coherent detection for such broadband communication systems strongly depends on the quality of channel estimation [2–5]. Fortunately, broadband multipath channels can be accurately estimated using adaptive filter techniques [6–10] such as the normalized least-mean-square (NLMS) algorithm, which has low complexity and can be easily implemented at the receiver. On the other hand, channel measurements have shown that broadband wireless multipath channels can often be described by only a small number of propagation paths with long delays [4, 11, 12]. Thus, a broadband multipath channel can be regarded as a sparse channel with only a few active dominant taps, while the other inactive taps are zero or close to zero. This inherent sparsity of the channel impulse response (CIR) can be exploited to improve the quality of channel estimation. However, such classical NLMS algorithms with a uniform step size across all filter coefficients have slow convergence when estimating sparse impulse response signals such as those in broadband sparse wireless multipath channels [11]. Consequently, corresponding algorithms have recently received significant attention in the context of compressed sensing (CS) [5, 12–14] and were already considered for channel estimation prior to the CS era [5, 12]. However, these CS channel estimation algorithms are sensitive to the noise in wireless multipath channels.

Inspired by the CS theory [12–14], several zero-attracting (ZA) algorithms have been proposed and investigated by combining the CS theory and the standard least-mean-square (LMS) algorithm for echo cancellation and system identification, which are known as the zero-attracting LMS (ZA-LMS) and reweighted ZA-LMS (RZA-LMS) algorithms, respectively [15]. Recently, this technique has been expanded to the NLMS algorithm and other adaptive filter algorithms to improve their convergence speed in a sparse environment [9, 16–18]. However, these approaches are mainly designed for nonproportionate adaptive algorithms. On the other hand, to utilize the advantages of the NLMS algorithm, such as stable performance and low complexity, the proportionate normalized least-mean-square (PNLMS) algorithm has been proposed and studied to exploit the sparsity in nature [19] and has been applied to echo cancellation in telephone networks. Although the PNLMS algorithm can utilize the sparsity characteristics of a sparse signal and obtain faster convergence at the initial stage by assigning independent magnitudes to the active taps, the convergence speed is reduced by even more than that of the NLMS algorithm for the inactive taps after the active taps converge. Consequently, several algorithms have been proposed to improve the convergence speed of the PNLMS algorithm [20–27], which include the use of the *l*_1_-norm technique and a variable step size. Although these algorithms have significantly improved the convergence speed of the PNLMS algorithm, they still converge slowly after the active taps converge. In addition, some of them are inferior to the NLMS and PNLMS algorithms in terms of the steady-state error when the sparsity decreases. From these previously proposed sparse signal estimation algorithms, we know that the ZA algorithms mainly exert a penalty on the inactive channel taps through the integration of the *l*_1_-norm constraint into the cost function of the standard LMS algorithms to achieve better estimation performance, while the PNLMS algorithm updates each filter coefficient with an independent step size, which improves the convergence of the active taps.

Motivated by the CS theory [13, 14] and ZA technique [15–18], we propose an *l*_*p*_-norm-constrained PNLMS (LP-PNLMS) algorithm that incorporates the *l*_*p*_-norm into the cost function of the PNLMS algorithm, resulting in an improved proportionate adaptive algorithm. The difference between the proposed LP-PNLMS algorithm and the ZA algorithms is that the gain-matrix-weighted *l*_*p*_-norm is used in our proposed LP-PNLMS algorithm instead of the general *l*_1_-norm to expand the application of ZA algorithms [15]. Also, this integration is equivalent to adding a zero attractor in the iterations of the PNLMS algorithm to obtain the benefits of both the PNLMS and ZA algorithms. Thus, our proposed LP-PNLMS algorithm can achieve fast convergence at the initial stage for the active taps. After the convergence of these active taps, the ZA technique in the LP-PNLMS algorithm acts as another force to attract the inactive taps to zero to arrest the slow convergence of the PNLMS algorithm. Furthermore, our proposed LP-PNLMS algorithm achieves a lower mean square error than the PNLMS algorithm and its related improved algorithms, such as the improved PNLMS (IPNLMS) [20] and *μ*-law PNLMS (MPNLMS) [21] algorithms. In this study, our proposed LP-PNLMS algorithm is verified over a sparse multipath channel by comparison with the NLMS, PNLMS, IPNLMS, and MPNLMS algorithms. The simulation results demonstrate that the LP-PNLMS algorithm achieves better channel estimation performance in terms of both convergence speed and steady-state behavior for sparse channel estimation.

The remainder of this paper is organized as follows.  Section 2 briefly reviews the standard NLMS, PNLMS, and improved PNLMS algorithms, including the IPNLMS and MPNLMS algorithms. In Section 3, we describe in detail the proposed LP-PNLMS algorithm, which employs the Lagrange multiplier method. In Section 4, the estimation performance of the proposed LP-PNLMS algorithm is verified over sparse channels and compared with other commonly used algorithms. Finally, this paper is concluded in Section 5.

## 2. Related Channel Estimation Algorithms

### 2.1. Normalized Least-Mean-Square Algorithm

**Figure 1:**
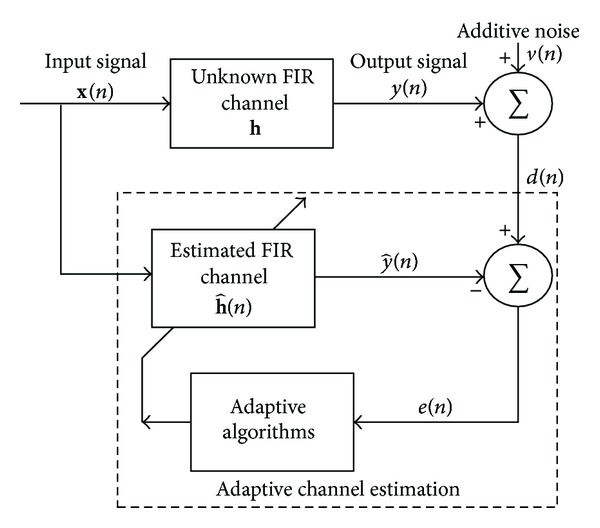
Typical sparse multipath communication system.

In this section, we first consider the sparse multipath communication system shown in Figure 1 to discuss the channel estimation algorithms. The input signal **x**(*n*) = [*x*(*n*), *x*(*n* − 1),…, *x*(*n* − *N* + 1)]^*T*^ containing the *N* most recent samples is transmitted over a finite impulse response (FIR) channel with channel impulse response (CIR) **h** = [*h*_0_, *h*_1_,…, *h*_*N*−1_]^*T*^, where (·)^*T*^ denotes the transposition operation. Then the output signal of the channel is written as follows:

(1) $$\begin{array}{c} y\left(n\right)=h^{T}x\left(n\right), \end{array}$$

where **h** is a sparse channel vector with *K* dominant active taps whose magnitudes are larger than zero and (*N* − *K*) inactive taps whose magnitudes are zero or close to zero with *K* ≪ *N*. To estimate the unknown sparse channel **h**, an NLMS algorithm uses the input signal **x**(*n*), the output signal *y*(*n*), and the instantaneous estimation error *e*(*n*), which is given by

(2) $$\begin{array}{c} e\left(n\right)=d\left(n\right)-{\hat{h}}^{T}\left(n\right)x\left(n\right), \end{array}$$

where $\hat{h}(n)$ is the NLMS adaptive channel estimator at instant *n*, *d*(*n*) = *y*(*n*) + *v*(*n*), and *v*(*n*) is an additive noise at the receiver. The update function of the NLMS channel estimation algorithm is expressed as

(3) $$\begin{array}{c} \hat{h}\left(n+1\right)=\hat{h}\left(n\right)+\mu_{\text{NLMS}}\frac{e\left(n\right)x\left(n\right)}{x^{T}\left(n\right)x\left(n\right)+\delta_{\text{NLMS}}}, \end{array}$$

where *μ*_NLMS_ is the step size with 0 < *μ*_NLMS_ < 2 and *δ*_NLMS_ is a small positive constant used to avoid division by zero.

### 2.2. Proportionate Normalized Least-Mean-Square Algorithm

The PNLMS algorithm, which is an NLMS algorithm improved by the use of a proportionate technique, has been proposed for sparse system identification and echo cancellation. In this algorithm, each tap is assigned an individual step size, which is obtained from the previous estimation of the filter coefficient. According to the gain allocation rule in this algorithm, the greater the magnitude of the tap, the larger the step size assigned to it, and hence the active taps converge quickly. The update function of the PNLMS algorithm [19] is described by the following equation with reference to Figure 1:

(4) $$\begin{array}{c} \hat{h}\left(n+1\right)=\hat{h}\left(n\right)+\mu_{\text{PNLMS}}\frac{e\left(n\right)G\left(n\right)x\left(n\right)}{x^{T}\left(n\right)G\left(n\right)x\left(n\right)+\delta_{\text{PNLMS}}}. \end{array}$$

Here, **G**(*n*), which denotes as the gain matrix, is a diagonal matrix that modifies the step size of each tap, *μ*_PNLMS_ is the global step size of the PNLMS algorithm, and *δ*_PNLMS_ = *δ*_*x*_^2^/*N* is a regularization parameter to prevent division by zero at the initialization stage, where *δ*_*x*_^2^ is the power of the input signal **x**(*n*). In the PNLMS algorithm, the gain matrix **G**(*n*) is given by

(5) $$\begin{array}{c} G\left(n\right)=\mathrm{diag}\left(g_{0}\left(n\right),g_{1}\left(n\right),\ldots ,g_{N-1}\left(n\right)\right), \end{array}$$

where the individual gain *g*_*i*_(*n*) is defined as

(6) $$\begin{array}{c} g_{i}\left(n\right)=\frac{\gamma_{i}\left(n\right)}{\sum_{i=0}^{N-1}\gamma_{i}\left(n\right)},\;0\leq i\leq N-1 \end{array}$$

with

(7) γi(n)=max⁡[ρgmax⁡[δp,|h^0(n)|,|h^1(n)|,…,|h^N−1(n)|],|h^i(n)|],

where the parameters *δ*_*p*_ and *ρ*_*g*_ are positive constants with typical values of *δ*_*p*_ = 0.01 and *ρ*_*g*_ = 5/*N*. *δ*_*p*_ is used to regularize the updating at the initial stage when all the taps are initialized to zero, and *ρ*_*g*_ is used to prevent ${\hat{h}}_{i}(n)$ from stalling when it is much smaller than the largest coefficient.

### 2.3. Improved Proportionate Normalized Least-Mean-Square Algorithms

#### 2.3.1. IPNLMS Algorithm

The IPNLMS algorithm is a type of PNLMS algorithm used to improve the convergence speed of the PNLMS algorithm. It is a combination of the PNLMS and NLMS algorithms with the relative significance of each coefficient controlled by a factor *α*. The IPNLMS algorithm [20] adopts the *l*_1_-norm to enable the smooth selection of (7), and the update equation of the IPNLMS algorithm is expressed as

(8) $$\begin{array}{c} \hat{h}\left(n+1\right)=\hat{h}\left(n\right)-\mu_{\text{IPNLMS}}\frac{e\left(n\right)K\left(n\right)x\left(n\right)}{x^{T}\left(n\right)K\left(n\right)x\left(n\right)+\delta_{\text{IPNLMS}}}, \end{array}$$

where **K**(*n*) = diag⁡(*k*_0_(*n*), *k*_1_(*n*),…, *k*_*N*−1_(*n*)) is a diagonal matrix used to adjust the step size of the IPNLMS algorithm, where

(9) kj(n)=1−α2N+(1+α)|h^j(n)|2||h^(n)||1+ɛ,0≤j≤N−1

for a small positive constant *ɛ* and −1 ≤ *α* ≤ 1. At the initial stage, the step size is multiplied by (1 − *α*)/2*N*, since all the filter coefficients are initialized to zero. Thus, in the IPNLMS algorithm, a regularization parameter *δ*_IPNLMS_ is introduced, which is given by

(10) $$\begin{array}{c} \delta_{\text{IPNLMS}}=\frac{1-\alpha }{2N}\delta_{\text{NLMS}}. \end{array}$$

We can see that the IPNLMS is identical to the NLMS algorithm for *α* = −1, while the IPNLMS behaves identically to the PNLMS algorithm when *α* = 1. In practical engineering applications, a suitable value for *α* is 0 or −0.5.

#### 2.3.2. MPNLMS Algorithm

The *μ*-law PNLMS algorithm (MPNLMS) is another enhancement of the PNLMS algorithm that utilizes the logarithm of the magnitudes of the filter coefficients instead of using the magnitudes directly in the PNLMS algorithm [21]. The update equation is the same as that in the PNLMS algorithm given by (4). In the MPNLMS algorithm,

(11) γi(n)=max⁡[ρgmax⁡[δpF(|h^0(n)|),F(|h^1(n)|),…,F(|h^N−1(n)|)],F(|h^i(n)|)],

where

(12) $$\begin{array}{c} F\left(\left|{\hat{h}}_{i}\left(n\right)\right|\right)=\log\left(1+\vartheta \left|{\hat{h}}_{i}\left(n\right)\right|\right), \end{array}$$

where *ϑ* is a large positive constant related to the estimation accuracy requirement, typically *ϑ* = 1000.

## 3. Proposed LP-PNLMS Algorithm

In this section, we propose an LP-PNLMS algorithm by incorporating the *l*_*p*_-norm into the cost function of the PNLMS algorithm to create a zero attractor, making it a type of ZA algorithm. The difference between the LP-PNLMS algorithm and general ZA algorithms is that the gain-matrix-weighted *l*_*p*_-norm is taken into account in designing the zero attractor. On the other hand, the proposed LP-PNLMS algorithm is based on the commonly used PNLMS algorithm, which is also a sparse channel estimation algorithm and can improve the convergence for the active taps. Regarding channel estimation, the purpose of the LP-PNLMS algorithm is to minimize

(13) (h^(n+1)−h^(n))TG−1(n) ×(h^(n+1)−h^(n))+γLP||G−1(n)h^(n+1)||psubject tod(n)−h^T(n+1)x(n)=0,

where **G** ^−1^(*n*) is the inverse of the gain matrix **G**(*n*) in the PNLMS algorithm, *γ*_LP_ > 0 is a very small constant used to balance the estimation error and the sparse *l*_*p*_-norm penalty of $\hat{h}(n+1)$, ||·||_*p*_ is the *p*-norm defined as ${||\hat{h}||}_{p}={\left(\sum_{i}{\hat{h}}_{i}^{p}\right)}^{1/p}$, and 0 ≤ *p* ≤ 1. Note that in (13), we introduce an *l*_*p*_-norm penalty to $\hat{h}(n+1)$ after scaling the gain matrix by **G** ^−1^(*n*), which is different from the previously proposed ZA LMS algorithms.

To minimize (13), the Lagrange multiplier method is adopted, and the cost function *J*_LP_(*n* + 1) of the proposed LP-PNLMS algorithm is expressed as

(14) JLP(n+1)=(h^(n+1)−h^(n))TG−1(n)×(h^(n+1)−h^(n))+γLP||G−1h^(n+1)||p+λ(d(n)−h^T(n+1)x(n)),

where *λ* is the Lagrange multiplier.

By calculating the gradient of the cost function *J*_LP_(*n* + 1) of the LP-PNLMS algorithm and assuming $\hat{h}(n+1)=\hat{h}(n)$ in the steady stage, we have

(15) $$\begin{array}{c} \frac{\partial J_{\text{LP}}\left(n+1\right)}{\partial \hat{h}\left(n+1\right)}=0,\;\;\frac{\partial J_{\text{LP}}\left(n+1\right)}{\partial \lambda }=0, \end{array}$$

(16) $$\begin{array}{c} \hat{h}\left(n+1\right)=\hat{h}\left(n\right)+\lambda G\left(n\right)x\left(n\right)-\gamma_{\text{LP}}\frac{{||\hat{h}(n)||}_{p}^{1-p}\underset{}{\mathrm{sgn}}\left(\hat{h}\left(n\right)\right)}{{\left|\hat{h}(n)\right|}^{1-p}}. \end{array}$$

In practice, we need to introduce a small positive constant into the final term in (16) to cope with the situation that an entry of $\hat{h}(n)$ approaches zero, which is the case for a sparse CIR at initialization. Then the update equation (16) of the LP-PNLMS algorithm is modified to

(17) $$\begin{array}{c} \hat{h}\left(n+1\right)=\hat{h}\left(n\right)+\lambda G\left(n\right)x\left(n\right)-\gamma_{\text{LP}}\frac{{||\hat{h}(n)||}_{p}^{1-p}\underset{}{\mathrm{sgn}}\left(\hat{h}\left(n\right)\right)}{{\left|\hat{h}(n)\right|}^{1-p}+{ɛ}_{p}}, \end{array}$$

where *ɛ*_*p*_ is a small value to prevent division by zero. By multiplying both sides of (17) by **x** ^*T*^(*n*), we obtain

(18) xT(n)h^(n+1)=xT(n)h^(n)+λxT(n)G(n)x(n)−γLPxT(n)||h^(n)||p1−psgn⁡(h^(n))|h^(n)|1−p+ɛp.

From (2), (15), and (17), we obtain

(19) e(n)=−γLPxT(n)||h^(n)||p1−psgn⁡(h^(n))|h^(n)|1−p+ɛp+λxT(n)G(n)x(n).

Then, the Lagrange multiplier *λ* is given as follows by solving (19):

(20) λ=(e(n)+γLPxT(n)||h^(n)||p1−psgn⁡(h^(n))|h^(n)|1−p+ɛp)×(xT(n)G(n)x(n))−1.

Substituting (20) into (17), we have

(21) h^(n+1)=h^(n)−γLP||h^(n)||p1−psgn⁡(h^(n))|h^(n)|1−p+ɛp +((e(n)+γLPxT(n)||h^(n)||p1−psgn⁡(h^(n))|h^(n)|1−p+ɛp)×(xT(n)G(n)x(n))−1)G(n)x(n)=h^(n)+e(n)G(n)x(n)xT(n)G(n)x(n) −γLP{I−G(n)x(n)xT(n)xT(n)G(n)x(n)} ×||h^(n)||p1−psgn⁡(h^(n))|h^(n)|1−p+ɛp.

It was found that the magnitudes of the elements in the matrix **G**(*n*)**x**(*n*)**x** ^*T*^(*n*){**x** ^*T*^(*n*)**G**(*n*)**x**(*n*)}^−1^ are much smaller than 1 for broadband multipath channel estimation. Therefore, the update equation (21) of the proposed LP-PNLMS algorithm is rewritten as

(22) h^(n+1)=h^(n)+e(n)G(n)x(n)xT(n)G(n)x(n)−γLP||h^(n)||p1−psgn⁡(h^(n))|h^(n)|1−p+ɛp.

Here, we neglect the effects of the matrix **G**(*n*)**x**(*n*)**x** ^*T*^(*n*){**x** ^*T*^(*n*)**G**(*n*)**x**(*n*)}^−1^ and assume that the filter order is large. Similarly to the PNLMS algorithm, a step size *μ*_LP_ is introduced to balance the convergence speed and the steady-state error of the proposed LP-PNLMS algorithm, and a small positive constant *ɛ*_LP_ = *δ*_*x*_^2^/*N* is employed to prevent division by zero. Thus, the update function (22) can be modified to

(23) h^(n+1)=h^(n)+μLPe(n)G(n)x(n)xT(n)G(n)x(n)+ɛLP −ρLP||h^(n)||p1−psgn⁡(h^(n))|h^(n)|1−p+ɛp=h^(n)+μLPe(n)G(n)x(n)xT(n)G(n)x(n)+ɛLP−ρLPT(n),

where *ρ*_LP_ = *μ*_LP_ *γ*_LP_ and $T(n)={||\hat{h}(n)||}_{p}^{1-p}{\mathrm{sgn}}_{}(\hat{h}(n)){\left\{{\left|\hat{h}(n)\right|}^{1-p}+{ɛ}_{p}\right\}}^{-1}$. Comparing the update function (23) of the proposed LP-PNLMS algorithm with the update function (4) of the PNLMS algorithm, we see that our proposed LP-PNLMS algorithm has the additional term *γ*_LP_ **T**(*n*), also defined as the zero attractor, which attracts the small channel taps to zero with high probability. Moreover, the ZA strength of this zero attractor is controlled by *ρ*_LP_. In other words, in our proposed LP-PNLMS algorithm, the gain matrix **G**(*n*) assigns a large step size to the active channel taps of the sparse channel, while the zero attractor mainly exerts the *l*_*p*_-penalty on the inactive taps whose taps are zero or close to zero. Thus, our proposed LP-PNLMS algorithm can further improve the convergence speed of the PNLMS algorithm after the convergence of the large active taps.

## 4. Results and Discussions

**Figure 2:**
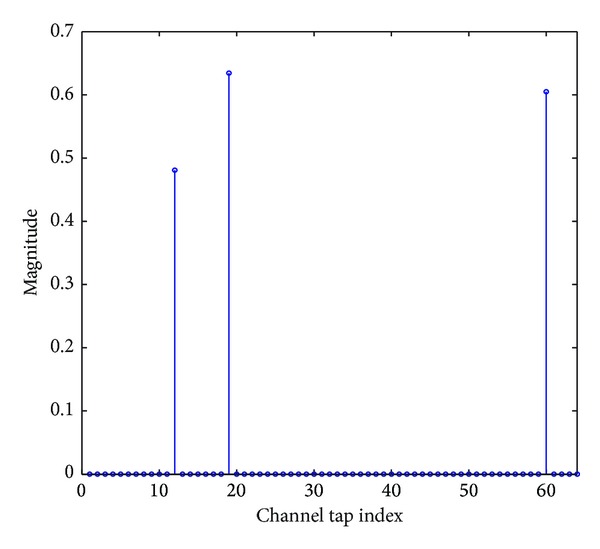
Typical sparse multipath channel.

In this section, we present the results of computer simulations carried out to illustrate the channel estimation performance of the proposed LP-PNLMS algorithm over a sparse multipath communication channel and compare it with those of the previously proposed IPNLMS, MPNLMS, PNLMS, and NLMS algorithms. Here, we consider a sparse channel **h** whose length *N* is 64 or 128 and whose number of dominant active taps *K* is set to three different sparsity levels, namely, *K* = 2, 4 and 8, similar to previous studies [6, 22, 25, 26]. The dominant active channel taps are obtained from a Gaussian distribution with ||**h**||_2_^2^ = 1, and the positions of the dominant channel taps are randomly spaced along the length of the channel. The input signal **x**(*n*) of the channel is a Gaussian random signal while the output of the channel is corrupted by an independent white Gaussian noise *v*(*n*). An example of a typical sparse multipath channel with a channel length of *N* = 64 and a sparsity level of *K* = 3 is shown in Figure 2. In the simulations, the power of the received signal is *E*_*b*_ = 1, while the noise power is given by *δ*_*v*_^2^ and the signal-to-noise ratio is defined as SNR = 10log⁡(*E*_*b*_/*δ*_*v*_^2^). In all the simulations, the difference between the actual and estimated channels based on the sparsity-aware algorithms and the sparse channel mentioned above is evaluated by the MSE defined as follows:

(24) $$\begin{array}{c} \text{MSE}\left(n\right)=10\;\log_{10}\text{E}\left\{{||h-\hat{h}(n)||}_{2}^{2}\right\}\left(\text{dB}\right). \end{array}$$

In these simulations, the simulation parameters are chosen to be *μ*_NLMS_ = *μ*_PNLMS_ = *μ*_IPNLMS_ = *μ*_LP_ = 0.5, *δ*_NLMS_ = 0.01, *ɛ* = 0.001, *α* = 0, *ɛ*_*p*_ = 0.05, *ρ*_LP_ = 1 × 10^−5^, *δ*_*p*_ = 0.01, *ρ*_*g*_ = 5/*N*, *ϑ* = 1000, *p* = 0.5, and SNR = 30 dB. When we change one of these parameters, the other parameters remain constant.

### 4.1. Estimation Performance of the Proposed LP-PNLMS Algorithm

#### 4.1.1. Effects of Parameters on the Proposed LP-PNLMS Algorithm

**Figure 3:**
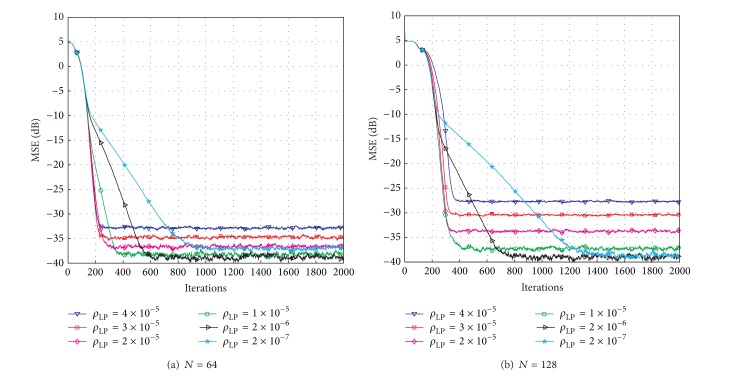
Effects of *ρ*_LP_ on the proposed LP-PNLMS algorithm.

**Figure 4:**
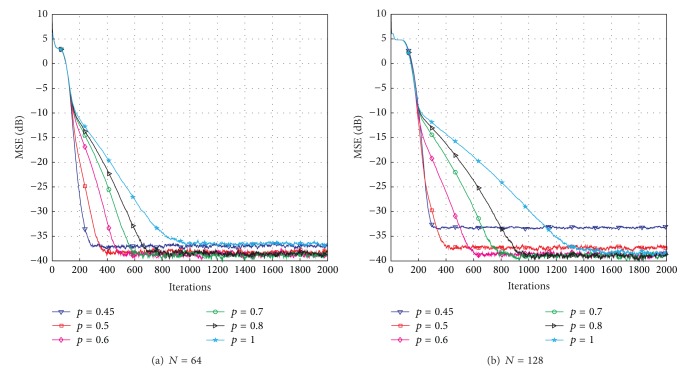
Effects of *p* on the proposed LP-PNLMS algorithm.

In the proposed LP-PNLMS algorithm, there are two extra parameters, *p* and *ρ*_LP_, compared with the PNLMS algorithm, which are introduced to design the zero attractor. Next, we show how these two parameters affect the proposed LP-PNLMS algorithm over a sparse channel with *N* = 64 or 128 and *K* = 4. The simulation results for different values of *ρ*_LP_ and *p* are shown in Figures 3 and 4, respectively. From Figure 3(a), we can see that the steady-state error of the LP-PNLMS algorithm decreases with decreasing *ρ*_LP_ when *ρ*_LP_ ≥ 2 × 10^−6^, while it increases again when *ρ*_LP_ is less than 2 × 10^−6^. Furthermore, the convergence speed of the LP-PNLMS algorithm rapidly decreases when *ρ*_LP_ is less than 1 × 10^−5^. This is because a small *ρ*_LP_ results in a low ZA strength, which consequently reduces the convergence speed. In the case of *N* = 128 shown in Figure 3(b), we observe that both the convergence speed and the steady-state performance are improved with decreasing *ρ*_LP_ for *ρ*_LP_ ≥ 1 × 10^−5^. When *ρ*_LP_ < 1 × 10^−5^, the convergence speed of the LP-PNLMS algorithm decreases while the steady-state error remains constant.

Figure 4 demonstrates the effects of the parameter *p*. We can see from Figure 4(a) that the convergence speed of the proposed LP-PNLMS algorithm rapidly decreases with increasing *p* for *N* = 64. Moreover, the steady-state error is reduced with *p* ranging from 0.45 to 0.5, while it remains constant for *p* = 0.6, 0.7, and 0.8. However, the steady-state performance for *p* = 1 is inferior to that for *p* = 0.8. This is because the proposed LP-PNLMS algorithm is an *l*_1_-norm-penalized PNLMS algorithm, which cannot distinguish between active taps and inactive taps, reducing its convergence speed and steady-state performance. When *N* = 128, as shown in Figure 4(b), the steady-state performance is improved as *p* increases from 0.45 to 0.6. Thus, we should carefully select the parameters *ρ*_LP_ and *p* to balance the convergence speed and steady-state performance for the proposed LP-PNLMS algorithm.

#### 4.1.2. Effects of Sparsity Level on the Proposed LP-PNLMS Algorithm

**Figure 5:**
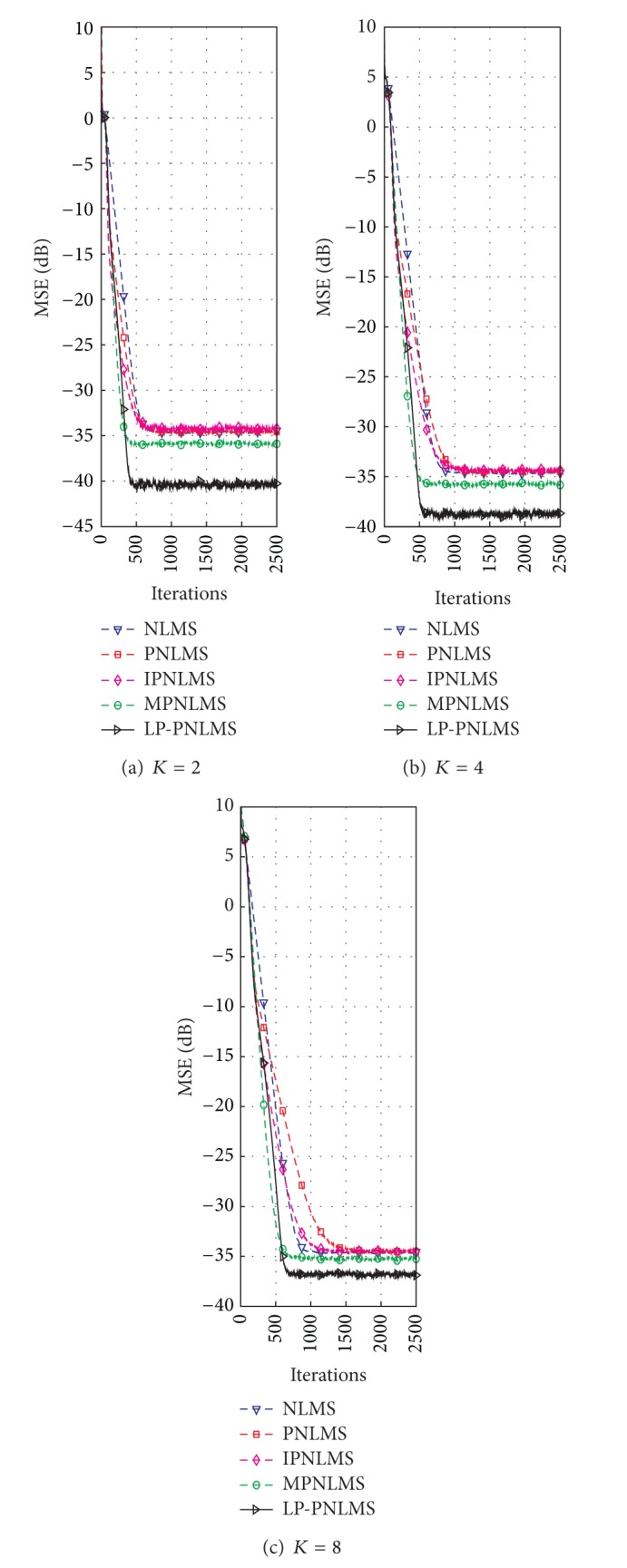
Effects of sparsity on the proposed LP-PNLMS algorithm for *N* = 64.

**Figure 6:**
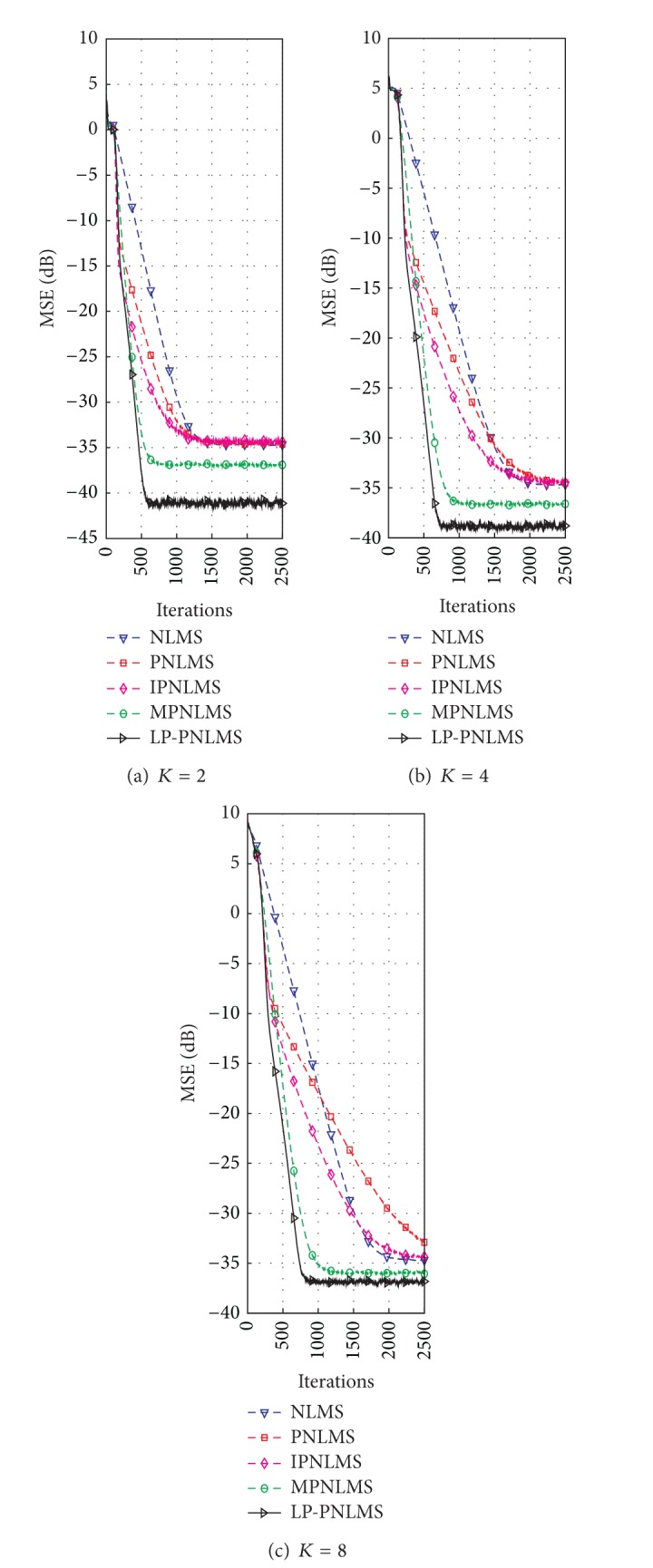
Effects of sparsity on the proposed LP-PNLMS algorithm for *N* = 128.

On the basis of the results discussed in Section 4.1.1 for our proposed LP-PNLMS algorithm, we choose *p* = 0.5 and *ρ*_LP_ = 1 × 10^−5^ to evaluate the channel estimation performance of the LP-PNLMS algorithm over a sparse channel with different channel lengths of *N* = 64 and 128, for which the obtained simulation results are given in Figures 5 and 6, respectively. From Figure 5, we see that our proposed LP-PNLMS algorithm has the same convergence speed as the PNLMS algorithm at the initial stage. The proposed LP-PNLMS algorithm converges faster than the PNLMS algorithm as well as the IPNLMS and NLMS algorithms for all sparsity levels *K*, while its convergence is slightly slower than that of the MPNLMS algorithm before it reaches a steady stage. However, the proposed LP-PNLMS algorithm has the smallest steady-state error for *N* = 64. When *N* = 128, we see from Figure 6 that our proposed LP-PNLMS algorithm not only has the highest convergence speed but also possesses the best steady-state performance. This is because with increasing sparsity, our proposed LP-PNLMS algorithm attracts the inactive taps to zero quickly and hence the convergence speed is significantly improved, while the previously proposed PNLMS algorithms mainly adjust the step size of the active taps and thus they only impact on the convergence speed at the early iteration stage. Additionally, we see from Figures 5 and 6 that both the convergence speed and the steady-state performance of all the PNLMS algorithms deteriorate when the sparsity level *K* increases for both *N* = 64 and 128. In particular, when *K* = 8, the convergence speeds of the PNLMS and IPNLMS algorithms are greater than that of the NLMS algorithm at the early iteration stage, while after this fast initial convergence, their convergence speeds decrease to less than that of the NLMS algorithm before reaching a steady stage. Furthermore, we observe that the MPNLMS algorithm is sensitive to the length *N* of the channel, and its convergence speed for *N* = 128 is less than that for *N* = 64 at the same sparsity level *K* and less than that of the proposed LP-PNLMS algorithm. Thus, we conclude that our proposed LP-PNLMS algorithm is superior to the previously proposed PNLMS algorithms in terms of both the convergence speed and the steady-state performance with the appropriate selection of the related parameters *p* and *ρ*_LP_. From the above discussion, we believe that the gain-matrix-weighted *l*_*p*_-norm method in the LP-PNLMS algorithm can be used to further improve the channel estimation performance of the IPNLMS and MPNLMS algorithms.

### 4.2. Computational Complexity

Finally, we discuss the computational complexity of the proposed LP-PNLMS algorithm and compare it with those of the NLMS, PNLMS, IPNLMS, and MPNLMS algorithms. Here, the computational complexity is the arithmetic complexity, which includes additions, multiplications, and divisions. The computational complexities of the proposed LP-PNLMS algorithm and the related PNLMS and NLMS algorithms are shown in Table 1.

From Table 1, we see that the computational complexity of our proposed LP-PNLMS algorithm is slightly higher than those of the MPNLMS and PNLMS algorithms, which is due to the calculation of the gradient of the *l*_*p*_-norm. Furthermore, the MPNLMS algorithm has an additional logarithm operation, which increases its complexity but is not included in the Table 1. However, the LP-PNLMS algorithm noticeably increases the convergence speed and significantly improves the steady-state performance of the PNLMS algorithm. In addition, it also has a higher convergence speed and lower steady-state error than the IPNLMS and MPNLMS algorithms when the channel length is large.

## 5. Conclusion

In this paper, we have proposed an LP-PNLMS algorithm to exploit the sparsity of broadband multipath channels and to improve both the convergence speed and steady-state performance of the PNLMS algorithm. This algorithm was mainly developed by incorporating the gain-matrix-weighted *l*_*p*_-norm into the cost function of the PNLMS algorithm, which significantly improves its convergence speed and steady-state performance. The simulation results demonstrated that our proposed LP-PNLMS algorithm, which has an acceptable increase in computational complexity, increases the convergence speed and reduces the steady-state error compared with the previously proposed PNLMS algorithms.

## Figures and Tables

**Table 1:** Computational complexity.

Algorithms	Additions	Multiplications	Divisions
NLMS	3*N*	3*N* + 1	1
PNLMS	4*N* + 3	6*N* + 3	*N* + 2
IPNLMS	4*N* + 7	5*N* + 5	*N* + 2
MPNLMS	5*N* + 3	7*N* + 3	*N* + 3
LP-PNLMS	4*N* + 4	9*N* + 4	2*N* + 2
